# Strengthening the Expanded Programme on Immunization in Africa: Looking beyond 2015

**DOI:** 10.1371/journal.pmed.1001405

**Published:** 2013-03-19

**Authors:** Shingai Machingaidze, Charles S. Wiysonge, Gregory D. Hussey

**Affiliations:** 1 Vaccines for Africa Initiative, Institute of Infectious Disease and Molecular Medicine, University of Cape Town, Cape Town, South Africa; 2 Division of Medical Microbiology, Department of Clinical Laboratory Sciences, University of Cape Town, Cape Town, South Africa

## Abstract

Shingai Machingaidze and colleagues reflect on the successes and shortfalls of the Expanded Programme on Immunization (EPI) in Africa, and the considerable challenges that must now be addressed to improve immunization systems.

**Summary Points**

- There have been significant improvements in the performance of the Expanded Programme on Immunization (EPI) in Africa since its inception in 1974. However, there exist wide inter- and intra-country differences.
- Successes such as the introduction of hepatitis B (HepB), *Haemophilus influenzae* type B (Hib), and meningococcal group A vaccines across the continent are milestones indicating growth and development in the right direction. Conversely polio and measles outbreaks, as well as high vaccine drop-out rates across the continent, indicate failures within the EPI system that require evidence-informed corrective interventions.
- With the 2015 deadline for the Millennium Development Goals (MDGs) approaching, it is necessary for Africa to take stock, critically assess its position, take ownership of the regional and country-specific problems, and develop precise strategies to overcome the challenges identified.
- There is need for increased immunisation systems strengthening, as many are plagued by weak infrastructure and shortage of skilled human resources. More affordable and adapted vaccines need to be made available.
- Increased political and financial commitments from African governments are key factors for both maintaining current achievements and making additional progress for EPI in Africa.

Immunisation is amongst the most cost-effective public health interventions for reducing global child morbidity and mortality [1],[2]. The global effort to use vaccination as a public health intervention began when the World Health Organization (WHO) launched the Expanded Programme on Immunization (EPI) in 1974. Over the years there have been several international efforts to increase EPI coverage, including Universal Childhood Immunisation [3]; the Global Alliance for Vaccines and Immunisation (GAVI) [4]; Millennium Development Goals (MDGs) [5]; the Global Immunisation Vision and Strategy (GIVS) [6]; and most recently, the Global Vaccine Action Plan (GVAP) [7]. These initiatives, coupled with specific regional efforts such as the WHO African Region's EPI strategic plans of action for the periods 2001–2005 and 2006–2009 [8], and the Reach Every District (RED) approach [9], as well as the efforts of national EPIs, have seen global coverage with three doses of the diphtheria-tetanus-pertussis vaccine by 12 months of age (DTP3) rise from 5% in 1974 to 85% in 2010 [10],[11]. However, sub-Saharan Africa reached only 77% DTP3 coverage in 2010 [10]. Although DTP3 is an acknowledged indicator of EPI performance, it is important to understand other EPI indicators in Africa. Here, we assess immunisation systems strengthening, accelerated disease control efforts, and the introduction of new and underutilised vaccines across Africa.

## Performance of Childhood Immunisation Programmes in Africa

### Immunisation Systems Strengthening

**Figure 1:**
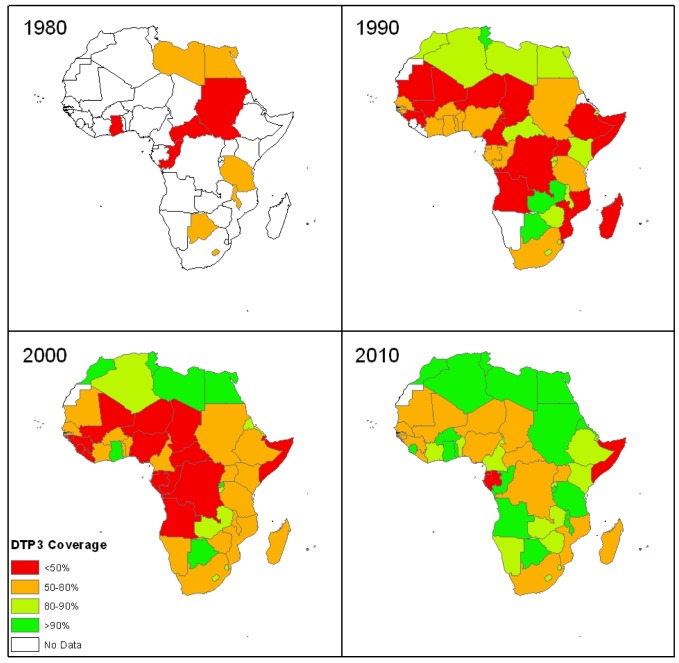
Colour-coded maps of Africa showing national coverage with the third dose of the diphtheria-tetanus-pertussis vaccine (DTP3) at the end of each decade since 1974. (Source of data: World Health Organization [10].)

Over the past four decades, extraordinary progress has been made in improving vaccination coverage in Africa (53 countries). According to WHO and the United Nations Children's Fund (UNICEF) coverage estimates, the number of countries with national DTP3 coverage of at least 80% increased from one (2%) in 1980, through 22 (42%) in 1990 and 17 (32%) in 2000, to 35 (67%) in 2010 (Figure 1) [10]. While 35 (67%) countries reported 80% national DTP3 coverage in 2010, only 16 (30%) countries reported at least 80% DTP3 coverage in 80% of their districts [10]. The DTP drop-out rate [((DTP1–DTP3)/DTP1)×100%] is also used for measuring EPI performance, where a DTP drop-out of less than 10% is considered good. Improvement in the proportion of countries having a DTP drop-out rate less than 10% was observed from 1974 till 1990, stagnating between 1990 and 2000, and then significantly increasing from 28% in 2000 to 60% in 2010 (*p* = 0.0013) [10].

In 2010, one-fifth of African countries were yet to implement use of auto-disable syringes as well as adequate safety and waste disposal measures [10],[12]. Cold chain management in resource-poor settings, where electricity is non-existent or erratic, coupled with a lack of adequate trained staff to administer vaccines present major challenges in most African countries [13]. Furthermore, of those children who do receive the vaccines, many receive them late or at inappropriate timing and likely receive sub-optimal disease protection [14]–[16].

Improvements in immunisation spending in most African countries have predominantly been due to donor funds. In 2005, 49% (26/53) of countries reported that at least half of the costs of their routine vaccines were funded by government [10]. However, of those that established line items in their national budgets for rountine vaccines, over a third of them did not fund them, and those that had drawn financial plans did not utilise them to the degree expected [17]. Only 15% (8/53) of African countries reported on routine vaccines funded by government in 2010 [10].

The quality of immunisation data in many African countries is questionable. Various external evaluations have identified many inconsistencies in reported data suggesting that immunisation data monitoring remains weak in most African countries [18]–[20].

### Accelerated Disease Control

Poliomyelitis, measles, yellow fever, and neonatal tetanus remain at the forefront of disease control efforts in Africa. By 2010, 81% (27/33) of the countries at risk for yellow fever had introduced the vaccination into their EPI schedules [21], compared to 27% (9/33) in 2000 [10].

Between 2000 and 2002, the number of African countries endemic with the wild poliovirus (WPV) decreased from 12 to two, and new WPV cases declined by 89% from 1,863 to 208 [17],[22]. However, following the cessation of polio immunisation for 12 months in the northern Nigerian state of Kano beginning in September 2003, WPV was imported into 29 previously polio-free African countries [23]. Religious and political leaders urged parents not to immunise their children as they believed the vaccine was contaminated with anti-fertility, HIV, and cancerous agents [24]. Since 2010, intensified efforts around the continent have resulted in WPV cases in Africa dropping by 47% from 657 in 2010 to 350 in 2011 [25]. Despite these efforts, in 2011, Nigeria remained endemic, transmission had re-established in Angola, Chad, and the Democratic Republic of the Congo (DRC), and ten other countries reported importations [26].

While routine measles vaccination had been established in most African countries by 1980, low coverage and outbreaks resulted in the initiation of catch-up and follow-up supplementary immunisation activities (SIAs) and case-based surveillance in the 1990s. A total of 24 million children in seven African countries were first reached during SIAs between 1996 and 2000 [27]. Between 2000 and 2010, all except four African countries conducted at least one catch-up SIA for children aged 9 months to 15 years, leading to a 92% reduction in measles mortality in sub-Saharan Africa between 2000 and 2008 [28]–[30]. Thereafter, due to low measles-containing vaccine (MCV) coverage and suboptimal follow-up SIAs, 27 countries had confirmed measles outbreaks between 2009 and 2011 [31].

In 2000, two-thirds (18/27) of the countries accounting for 90% of global neonatal tetanus cases were in Africa [32]. Thereafter, neonatal tetanus elimination was validated in 20 (38%) African countries in 2005, increasing to 27 (51%) in 2010 [10].

### New and Underutilised Vaccines

There has been significant but slow progress in the introduction of new and underutilised vaccines in Africa. HepB and Hib vaccines were first licensed in the United States in 1981 and 1985, respectively. By 2005, 20 years later, the proportion of African countries having introduced HepB (70%) and Hib (26%) highlight the need to address underutilisation of available vaccines on the continent [10].

Although pneumonia and diarrhoea remain leading causes of child death [33], only seven African countries had introduced the rotavirus and/or pneumococcal conjugate (PCV) vaccines by 2010. These are “new vaccines” on the market that are costly and largely unaffordable for most African countries. Some progress is anticipated with an additional 16 countries and nine countries planning to introduce PCV and rotavirus vaccines, respectively, by 2013, with GAVI funding [10]. The Meningitis Vaccine Project (MVP) is a modern vaccine success story that saw the development of a new vaccine against meningococcal group A (the predominant cause of epidemic meningitis in the “meningitis belt”) being developed within 10 years. Negotiations between the MVP and partners around the world allowed for the development of this conjugate vaccine (MenAfriVac) in a low- and middle-income country (LMIC), India, at a cost of less than US$0.50/dose [34]. Within 2 years of licensure, six of the 25 countries at risk have already introduced MenAfriVac with GAVI support [33].

## What Are the Implications for African Countries?

It is estimated that 1.5 million children died globally from vaccine-preventable diseases (VPDs) for which there were available WHO pre-qualified vaccines in 2010 [35]. Approximately 19.3 million children did not receive DTP3 worldwide in 2010, with more than one-third of these children living in Africa [36]. While Africa has made remarkable improvements in immunisation services, this agenda remains largely unfinished with large numbers of children remaining unreached, unvaccinated, under-vaccinated, and still dying from VPDs. While many countries have introduced HepB and Hib vaccines, the majority have not yet introduced PCV and rotavirus vaccines, and will not have done so by 2015. This continued failure to meet agreed targets thus far suggests that general and country-specific challenges with regards to immunisation programmes in Africa have not been fully identified, understood, and/or addressed effectively [37].

## Paving the Way forward for an Improved EPI in Africa

With the 2015 deadline for the MDGs fast approaching [5], it is necessary for Africa to take stock, critically assess its position, take ownership of the regional and country-specific problems, and develop precise strategies to overcome the challenges identified. The annual cost per live birth for immunisation in LMICs is estimated to have increased from an average of US$6.00 in 2000 to US$25.00 in 2008 (due to HepB and Hib vaccines) and is likely to increase to US$58.00 if PCV and rotavirus vaccines are included [3]. Even with continued support from donors, political will as well as financial planning and commitment from African governments will be key factors for successful introduction and sustainability of new vaccines in EPI schedules in Africa. Issues of immunisation awareness, demand for immunisation, level of trust in health system, adequate human resources, access, timeliness of vaccinations, service delivery, poor infrastructure, and immunisation monitoring are among the many challenges faced by most African countries, all requiring evidence-based interventions [37]–[39]. Rwashana and colleagues from Uganda provide a good example of understanding the specific challenges and dynamics faced by an individual immunisation programme in the African context [40]. Identification of suitable and effective interventions to address identified problems within immunisation programmes is then of great importance to ensure best use of the limited resources available [41],[42]. Vaccine procurement and pricing strategies, vaccine adaptation to suit LMICs, as well as the establishment of vaccine production in LMICs remain essential components of helping to strengthen immunisation systems across Africa [13]. The polio boycott in northern Nigeria highlights the need for adequate communication about vaccines across Africa, the consequences of not urgently addressing concerns about perceived vaccine safety, as well as the need for prompt high-quality outbreak investigations [22]–[26].

We believe that in order for Africa to take advantage of the new decade of vaccines and extend the full benefits of immunisation to its citizens by 2020 and beyond, a critical assessment is a fundamental first step. The recently introduced GVAP is guided by six principles: country ownership, shared responsibility and partnership, equity, integration, sustainability, and innovation [7]. While this provides generalised strategies for attaining outlined goals, it is absolutely necessary for complementary tailor-made African approaches accompanied by robust monitoring and accountability frameworks.

## Conclusion

African countries must be commended for giant steps made in EPI performance. However, there exist wide inter- and intra-country differences, with large numbers of African children remaining unreached, unvaccinated, under-vaccinated, and still dying from VPDs. Immunisation systems strengthening is essential, as most are under-staffed with inadequate resources to function efficiently. Issues of vaccine supply, financing, and sustainability in Africa require urgent attention. Increased political and financial commitment from governments as well as coordinated national and continental evidence-informed efforts by all immunisation stakeholders are needed to both maintain current achievements and make additional progress for EPI in Africa. African leaders must be held accountable for meeting agreed country targets and honouring international commitments made.

## Abbreviations

DTP3: diphtheria-tetanus-pertussis third dose
EPI: Expanded Programme on Immunization
GAVI: Global Alliance for Vaccines and Immunisation
HepB: hepatitis B
Hib: *Haemophilus influenzae* type B
LMIC: low- and middle-income country
MCV: measles-containing vaccine
MDG: Millennium Development Goal
MVP: Meningitis Vaccine Project
PCV: pneumococcal conjugate vaccine
SIA: supplementary immunisation activity
UNICEF: United Nations Children's Fund
VPD: vaccine-preventable disease
WHO: World Health Organization
WPV: wild polio virus
